# Molar Ratio Effect of Sodium to Chloride Ions on the Electrochemical Corrosion of Alloy 600 and SA508 in HCl + NaOH Mixtures

**DOI:** 10.3390/ma13081970

**Published:** 2020-04-23

**Authors:** Do Haeng Hur, Jeoh Han, Jun Choi

**Affiliations:** 1 Korea Atomic Energy Research Institute, 989-111, Deadeok-daero, Yuseong-gu, Daejeon 34057, Korea; jeohhan@kaeri.re.kr (J.H.); jun@dsv.co.kr (J.C.); 2 Daesung Machinery Co., Ltd., 3Da-401, Sihwa Industrial Complex, 334, Gongdan 1-daero, Gyeonggi-do, Siheung 15106, Korea

**Keywords:** molar ratio index, steam generator, tubesheet, crevice chemistry, galvanic couple

## Abstract

This study aims to investigate the molar ratio effect of sodium to chloride ions on the corrosion of an Alloy 600 steam generator tube and an SA508 tubesheet material. The corrosion behavior was evaluated in solutions with three different molar ratios of sodium to chloride ions using a potentiodynamic polarization method. The corrosion potentials and corrosion rates of both the two materials were significantly decreased as the molar ratio increased from 0.1 to 10. Therefore, it is recommended that the molar ratio control to a value of 1 is beneficial only when the crevice chemistry has a low molar ratio with an acidic pH. The corrosion potentials and corrosion rates were little affected by the total sodium and chloride ion concentrations. SA508 acted as an anode and its corrosion rate was accelerated by galvanic coupling with Alloy 600.

## 1. Introduction

Stress corrosion cracking (SCC), intergranular attack (IGA) and pitting have been major degradation modes of Alloy 600 steam generator (SG) tubes in the secondary side water environments of pressurized water reactors (PWRs) [1,2,3,4]. Corrosion damage of the Alloy 600 tube materials is accelerated in both acidic and alkaline environments resulting from the impurity concentration processes. The formation of these corrosive environments is closely associated with local boiling within tube to tube support crevices and tube to tubesheet crevices in which sludge is accumulated [5,6]. The laboratory and field experience data have indicated that both IGA and SCC of Alloy 600 steam generator tubes are minimized at a near neutral pH [7,8]. Therefore, it is expected that maintaining a crevice pH near neutral reduces the corrosion damage of Alloy 600. 

Based on the above backgrounds, the molar ratio control program was developed by the Electric Power Research Institute (EPRI), of which the goal is to maintain the crevice chemistry at near neutral pH values [7]. A molar ratio index (MRI) for managing the secondary water chemistry in PWRs was also proposed as the following equation [7]:

(1) $$\mathrm{MRI}=\frac{Na+K}{Cl+excess\;SO_{4}}$$

To maintain a desired MRI, several methods has been implemented, such as sodium source reduction, chloride injection, and ion exchange resin manipulation [9]. This index was derived from a viewpoint of the corrosion behavior of SG tube material itself. SG tubes are equipped in a tubesheet by expanding the both end parts of the tubes within drilled-holes of the tubesheet. The SG tubing is made of nickel-based alloys, while the tubesheet is SA508 low alloy steels. Therefore, an SG tube and a tubesheet are galvanically contacted in the tube to tubesheet crevice region of a SG. In addition, corrosion of the tubesheet material induces denting damage of the tubes at the top of the tubesheet, accelerating SCC of the tubes [10,11]. Therefore, the corrosion behavior of the tubesheet material should also be considered in the application of the molar ratio control. In addition, since the MRI is a simple molar ratio of cations to anions, the total concentration of the cations and the anions is not considered. 

Potentiodynamic polarization tests provide useful information such as corrosion rate and susceptibility of materials to corrosion in aqueous solutions, as well as pitting corrosion. Furthermore, it has been reported that polarization behaviors of Alloy 600 and stainless steels are associated with IGA and SCC [12,13,14]. In this case, however, additional IGA and SCC tests should be performed to find a correlation at applied potentials selected from a polarization curve. In this work, an electrochemical polarization method was used to survey the effect of the MRI on the polarization responses of an Alloy 600 SG tube and an SA508 tubesheet material at approximately 25 °C and to provide bases for comparison with the results at higher temperatures, which will be obtained in the next phase. The effect of impurity concentration at a constant MRI was also evaluated.

## 2. Experimental Methods

### 2.1. Specimen and Solution Preparation

Alloy 600 and SA508 were used as a tube material and a tubesheet material of a SG, respectively. Chemical compositions of the materials are given in Table 1 and Table 2. Alloy 600 had an average grain size of about 48.9 mm with chromium carbides at grain boundaries, which satisfied the EPRI specification [15]. SA508 was heat-treated according to the American Society of Mechanical Engineers (ASME) standard specification [16] and its microstructure was typical bainite. Because the material microstructure has a significant impact on corrosion behavior [17,18,19], all specimens were prepared from a single heat of each material.

Specimens were cut into a size of 10 × 5 × 1 mm^3^ for the electrochemical corrosion tests. They were ground using silicon carbide paper down to 1000-grit and then ultrasonically cleaned in acetone for 5 min.

To prepare a working electrode for the electrochemical test, an Alloy 600 specimen was spot-welded to an Alloy 600 wire, while an SA508 specimen was spot-welded to a pure iron wire. The lead wire was then shielded with a polytetrafluoroethylene (PTFE) tube for electrical insulation. A resin was coated around the spot-weld to prevent the test solution from penetrating into any remaining crevice there. After curing the resin, the specimen was ultrasonically cleaned in acetone for 1 min. When subtracting the weld-junction area, the surface area exposed to the solutions during the electrochemical tests was 1.28 cm^2^.

The MRIs of sodium ions to chloride ions in the test solutions were controlled to be 0.1, 1 and 10 by the addition of HCl and NaOH into demineralized water with the resistivity near 18 MΩ∙cm, as shown in Table 3. Any other chemical species were not included to simplify the Equation (1). The total ion concentrations of sodium and chloride ions were fixed to be 0.011 and 0.11 M at each MRI. Regardless of the total ion concentrations, the measured solution pH was dependent on the MRI and was about 2 at the MRI 0.1, 7 at the MRI 1, and 12 at the MRI 10.

### 2.2. Electrochemical Corrosion Test

Potentiodynamic polarization tests were performed in each solution at 25 °C by using a PAR273 potentiostat (EG&G Princeton Applied Research, Berwyn, PA, USA) with Power-Suite software (version 2.58, Ametek, Berwyn, PA, USA) and a conventional corrosion cell with three electrodes. A saturated calomel electrode was used as the reference electrode, and a platinum wire was used as the counter electrode. The test solutions were deaerated by bubbling ultra-high purity (99.999%) nitrogen gas at a rate of 300 mL/min. The open circuit potential (OCP) of a working electrode reached a stable value within 1 h. After that, the potential was scanned either to the positive direction for the anodic curve, or to the negative direction for the cathodic curve at a rate of 20 mV/min under continuous blowing of nitrogen. Each anodic and cathodic polarization curve was finally combined in one graph. The polarization curves were obtained at least three times to ensure their reproducibility using fresh specimens and solutions.

The corrosion current density (*i_corr_*) of the materials at the OCPs was calculated by using the Tafel extrapolation method of cathodic polarization curves. The galvanic corrosion potential and the galvanic current density between Alloy 600 and SA508 were determined by the application of the mixed potential theory.

## 3. Results and Discussion

**Figure 1:**
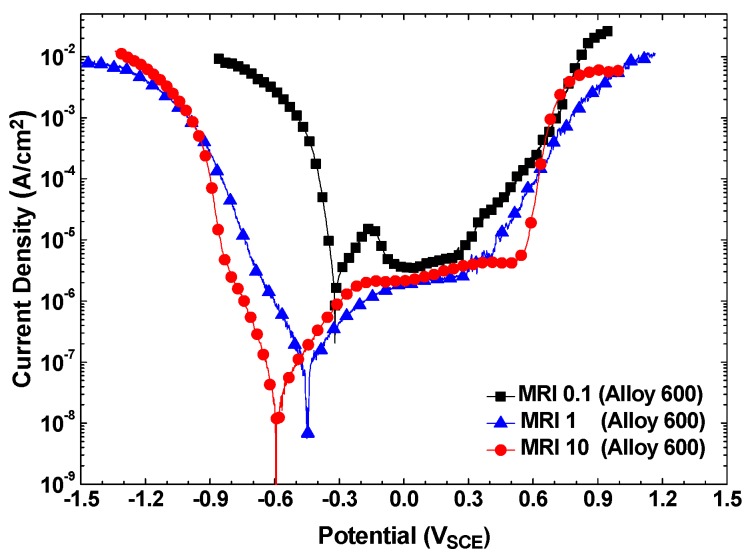
Polarization curves of Alloy 600 in the solutions of the MRI 0.1, 1 and 10 at a total sodium and chloride ion concentration of 0.011 M.

Figure 1 shows the potentiodynamic polarization behaviors of Alloy 600 in the solutions of MRI 0.1, 1, and 10 at a total sodium and chloride concentration of 0.011 M. The corrosion potentials (*E_corr_*) and corrosion rates (*i_corr_*) of Alloy 600 were significantly decreased as the MRI increased from 0.1 to 10. The active–passive transition appeared at the MRI 0.1, but the alloy was passivated without active dissolution at the MRI 1 and 10. 

**Figure 2:**
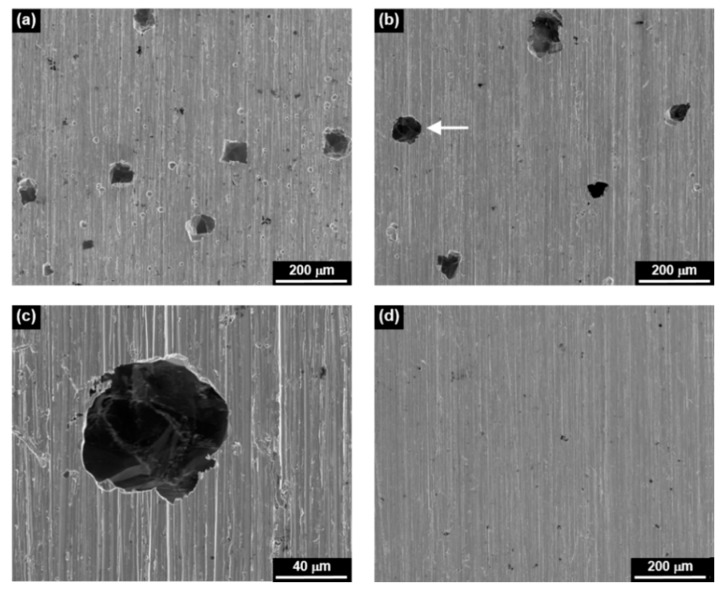
Scanning electron microscopy (SEM) micrographs showing the corroded surfaces of Alloy 600 after polarization scans at (**a**) the MRI 0.1, (**b**) the MRI 1, (**c**) magnification of the pit denoted by the white arrow in (**b**), and (**d**) the MRI 10.

Figure 2 shows the SEM micrographs of corroded surfaces after the anodic polarization scans. The surfaces exposed to the MRI 0.1 and 1 solutions showed extensive pitting, whereas the surface at the MRI 10 was uniformly corroded without pitting corrosion. Therefore, the transpassivity showing an abrupt increase of current at about 0.260 and 0.390 V in the solutions of the MRI 0.1 and 1, respectively, was due to pitting, whereas the current increase at 0.600 V in the solution of the MRI 10 was owing to oxygen evolution, the reaction of which can be expressed by the following equation [20]:

2H_2_O = O_2_ + 4H^+^ + 4e (2)

**Figure 3:**
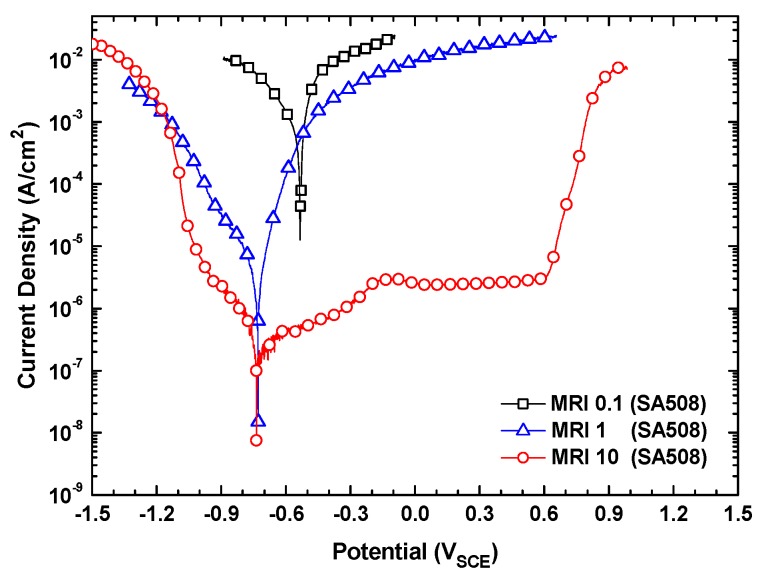
Polarization curves of SA508 in the solutions of the MRI 0.1, 1 and 10 at a total sodium and chloride ion concentration of 0.011 M.

As shown in Figure 3, the corrosion potentials of SA508 were also significantly decreased as the MRI increased from 0.1 to 10. SA508 actively dissolved at high corrosion rates without any passivation at the MRI 0.1 and 1, whereas the alloy showed the lowest corrosion rate with a passive behavior in a potential range of −0.190 to 0.600 V at the MRI 10. SA508 also showed an abrupt increase of current density due to oxygen evolution near a potential of 0.600 V at the MRI 10, as did Alloy 600.

**Figure 4:**
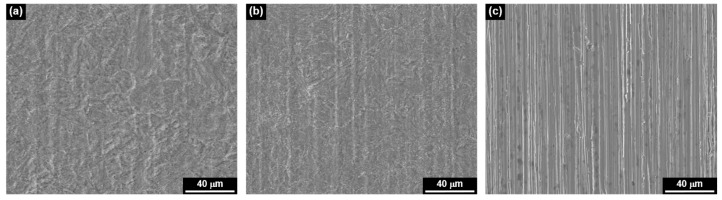
SEM micrographs showing the corroded surfaces of SA508 after polarization scans at (**a**) the MRI 0.1, (**b**) the MRI 1, and (**c**) the MRI 10.

Figure 4 shows the SEM micrographs of SA508 surfaces after the anodic polarization scans. The surface exposed to the MRI 0.1 was severely and uniformly corroded enough to dissolve out the grinding marks, which was made by emery paper during the surface finishing process. The surface at the MRI 1 was also corroded uniformly, but less severely than at the MRI 0.1. On the contrary, it can still clearly be seen the grinding marks on the surface exposed to the MRI 10, indicating that the anodic dissolution rate was very low in the MRI 10 solution. Therefore, the morphologies of these corroded surfaces were in good agreement with the polarization behaviors shown in Figure 3.

The important corrosion parameters from Figure 1 and Figure 3 are summarized in Table 4. Alloy 600 showed the highest corrosion rate at the MRI 0.1, while the corrosion rates at the MRIs 1 and 10 were nearly similar. In case of SA508, this alloy also showed the highest corrosion rate at the MRI 0.1, but the corrosion rate at the MRIs 10 was rather smaller than that at the MRI 1. Consequently, this result indicates that the molar ratio control method is beneficial only when the crevice chemistry has a low MRI and pH.

As shown in Table 1 and Table 2, Alloy 600 is a high-alloyed steel containing 15.7 wt.% Cr and 73.7 wt.% Ni. Thus, this alloy has an excellent resistance to corrosion in overall pH ranges from acidic to alkaline. Therefore, the difference between the corrosion rates (*i_corr_*) at the acidic MRI 0.1 and at the alkaline MRI 10 is not so large. However, SA508 is an iron-based steel containing only 0.23 wt.% Cr and 0.58 wt.% Mo and thus has a basically poor corrosion resistance, especially in acidic solutions. From Table 4, the corrosion rates (*i_corr_*) of Alloy 600 were always significantly lower than those of SA508 in all the test conditions. In addition, there was a significant decrease of *i_corr_* for SA508 at the MRI 10 in comparison with the MRI 1 as well as the MRI 0.1. The reason for this can be attributed to the fact that the solubility of magnetite is significantly dependent on the pH of a solution [21,22]. The solubility of magnetite at pH 3 is about 5 × 10^4^ times higher than that at pH 12 in water at 100 °C [21]. Therefore, the corrosion rate of SA508 increases significantly in low pH solutions (i.e., low MRI solutions) with a high solubility of magnetite because the corroding surface of the alloy cannot be protected by the magnetite film. Conversely, the alloy showed a passive behavior with a low corrosion current in a potential range of −0.190 to 0.600 V at the MRI 10 solution of pH 12, owing to a significantly low solubility of magnetite.

**Figure 5:**
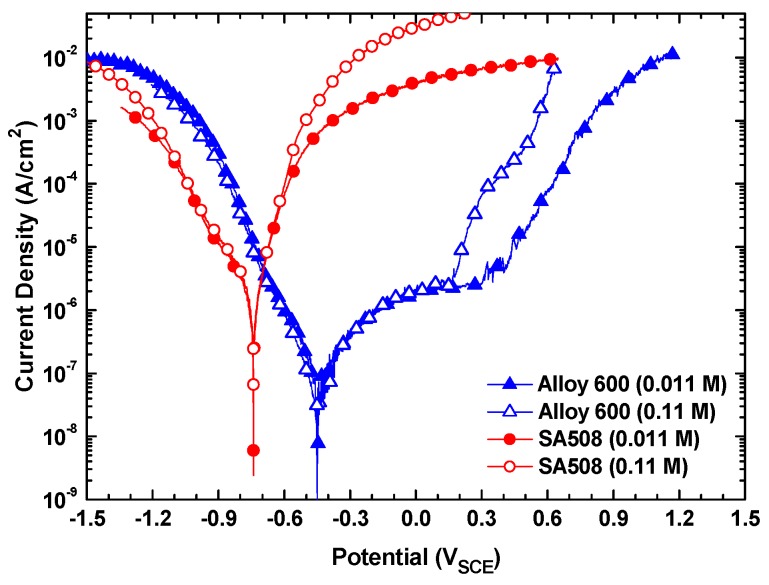
Polarization curves of Alloy 600 and SA508 in the 0.011 and 0.11 M solutions at a constant MRI 1.

Figure 5 shows the potentiodynamic polarization behaviors of Alloy 600 and SA508 in the solutions with total sodium and chloride ion concentration of 0.011 M and 0.11 M at a constant MRI 1. The corrosion potentials of Alloy 600 and SA508 were approximately −0.450 V and −0.730 V at both concentrations, respectively, indicating that the corrosion potentials of the two materials were not affected by a change in the total ion concentration. The cathodic and anodic current density of Alloy 600 was also little affected by an increase of the ion concentration from 0.011 M to 0.11 M. However, the pitting potentials of Alloy 600 decreased from 0.390 V in 0.011 M to 0.170 V in 0.11 M. In case of SA508, the polarization current density was nearly same in the solutions of 0.011 M and 0.11 M, when this alloy was polarized around the corrosion potential. The above results mean that the electrochemical corrosion behavior of these materials in a region near the corrosion potentials does not depend on the total sodium and chloride ion concentrations if the sodium to chloride molar ratio in a solution is the same. Similar behaviors were also observed at the MRI 0.1 and 10.

**Figure 6:**
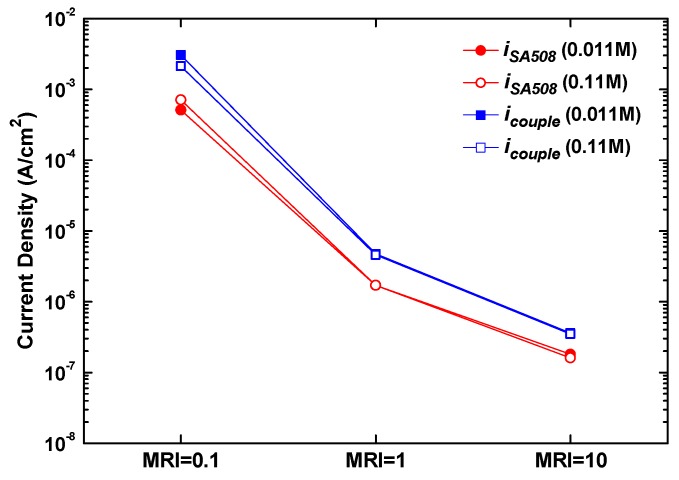
Effect of the MRIs on the galvanic corrosion rate of SA508 coupled to an equal area of Alloy 600.

From Figure 1, Figure 3, and Figure 5, it is clear that the corrosion potential of SA508 is always lower than that of Alloy 600 in each test condition. The anodic curve of SA508 also intersects with the cathodic curve of Alloy 600. This result demonstrates that SA508 is an anodic member of the galvanic couple and its corrosion rate is accelerated, when SA508 and Alloy 600 are electrically contacted. When the two materials are coupled in equal area, the galvanic current density (*i_couple_*) of SA508, acting as an anode, is determined at the intersection of the anodic curve of SA508 and the cathodic curve of Alloy 600. Figure 6 shows the effect of the MRIs on the galvanic corrosion of SA508, based on the polarization curves. Upon coupling to an equal area of Alloy 600, the current density (*i_couple_*) of the coupled SA508 was increased by about 2~6 times compared to that (*i_SA508_*) before coupling. However, the area of the tubesheet around a tube is much smaller than that of the tube in actual SGs, because the tubes are densely inserted into the tubesheet to increase the heat transfer area. Consequently, the corrosion rate of SA508 would be more accelerated by the effect of small anode (SA508) and large cathode (Alloy 600). In addition, the galvanic corrosion rate of SA508 was little changed by the total ion concentration at a fixed MRI as shown in Figure 6. This is because the polarization current density of the two materials was not affected by an increase in the total ion concentration from 0.011 M to 0.11 M, as shown in Figure 5. 

An SG tube can be slowly deformed by volume expansion of corrosion products due to corrosion of the tube support materials adjacent to and around the tube, which is called denting. The denting was attributed to concentration of chlorides and oxidants such as copper, in the crevices, leading to rapid corrosion of the tube support materials [2,23]. SG tubes are expanded into the tubesheet of SA508, a dissimilar metal. Therefore, based on the results obtained in this work, it is worth mentioning that corrosion of the tubesheet is accelerated by the galvanic coupling itself without concentration of chemical impurities in the crevices.

## 4. Conclusions

The electrochemical corrosion behavior of Alloy 600 and SA508 was investigated in solutions with three different molar ratios of sodium to chloride ions. The corrosion potentials and corrosion rates of both materials were significantly decreased as the molar ratio increased from 0.1 to 10. Therefore, it is expected that the molar ratio control method is beneficial only when the crevice chemistry has a low molar ratio with an acidic pH. The corrosion potentials and corrosion rates were little affected by the total sodium and chloride ion concentrations if the alloys were polarized not far from their corrosion potentials. Alloy 600 and SA508 acted as a cathode and an anode, respectively, when they were electrically coupled. Therefore, this result indicates that the corrosion rate of the SA508 tubesheet material is accelerated owing to the galvanic coupling effect itself without concentration of chemical impurities in the crevices.

## Figures and Tables

**Table 1:** Chemical composition of Alloy 600 material (wt.%).

C	Cr	Fe	Si	Mn	Ti	Al	Ni
0.02	15.7	10.0	0.1	0.3	0.1	0.06	Bal.

**Table 2:** Chemical composition of SA508 material (wt.%).

C	Si	Mn	P	S	Ni	Cr	Mo	Fe
0.199	0.051	1.52	0.005	0.006	0.987	0.232	0.582	Bal.

**Table 3:** Experimental conditions for the electrochemical corrosion tests.

Na^+^ (M)	Cl^−^ (M)	Total Concentration of Na^+^ and Cl^−^ (M)	MRI (Na^+^/Cl^−^)	pH
0.001	0.01	0.011	0.1	2
0.0055	0.0055	0.011	1	7
0.01	0.001	0.011	10	12
0.01	0.1	0.11	0.1	2
0.055	0.055	0.11	1	7
0.1	0.01	0.11	10	12

**Table 4:** Corrosion potentials and corrosion rates of Alloy 600 and SA508 obtained from the polarization tests.

MRI	Alloy 600		SA508	
	*E_corr_* (V_SCE_)	*i_corr_* (μA/cm^2^)	*E_corr_* (V_SCE_)	*i_corr_* (μA/cm^2^)
0.1	−0.314	3.2	−0.532	520
1	−0.446	0.088	−0.732	1.8
10	−0.593	0.064	−0.728	0.19
